# The Course of Self-Efficacy for Therapeutic Use of Self in Norwegian Occupational Therapy Students: A 10-Month Follow-Up Study

**DOI:** 10.1155/2018/2962747

**Published:** 2018-04-01

**Authors:** Kathrin Schwank, Tove Carstensen, Farzaneh Yazdani, Tore Bonsaksen

**Affiliations:** ^1^Europäische Fachhochschule, Rostock, Germany; ^2^Department of Neuromedicine and Movement Science, Norwegian University of Science and Technology, Trondheim, Norway; ^3^Faculty of Health and Life Sciences, Oxford Brookes University, Oxford, UK; ^4^Department of Occupational Therapy, Prosthetics and Orthotics, Faculty of Health Sciences, OsloMet–Oslo Metropolitan University, Oslo, Norway; ^5^Faculty of Health Studies, VID Specialized University, Sandnes, Norway

## Abstract

**Background:**

Occupational therapy students need to develop self-efficacy for managing the therapeutic relationship in practice. This study examined the 10-month trajectories of Norwegian students' self-efficacy for use of self.

**Methods:**

Eighty-nine students completed self-efficacy questionnaires related to the use of self after a workshop and at 3- and 10-month follow-up. Changes on the three outcome measures (self-efficacy for therapeutic mode use, for recognizing clients' interpersonal characteristics, and for managing interpersonal events) were analyzed with repeated measures ANOVA.

**Results:**

Across the follow-up period, the students improved their self-efficacy for therapeutic mode use (partial *η*^2^ = 0.44, *p* < 0.001), for recognizing clients' interpersonal characteristics (partial *η*^2^ = 0.81, *p* < 0.001), and for managing interpersonal events (partial *η*^2^ = 0.32, *p* < 0.001).

**Conclusion:**

The increased self-efficacy for use of self that was found at 3-month follow-up was maintained at 10-month follow-up. The results indicate that students may experience a boost in self-efficacy for therapeutic use of self after a brief workshop and that these changes can be sustained over time.

## 1. Introduction

The Intentional Relationship Model (IRM) is a conceptual framework for describing the therapeutic relationship and the therapeutic use of self in the context of occupational therapy practice. Introduced by Taylor [1], the model outlines the interrelated aspects of the client-therapist relationship and how the relationship may be used as a therapeutic means, with a potential impact on the client's occupational engagement.

The IRM posits four main variables [1, 2]. First, occupation is viewed as the core mechanism of change in occupational therapy. The therapist should consider the client's engagement in meaningful occupation as the primary goal. Second, the client should be understood with regard to his or her interpersonal characteristics, which are in part based on underlying personality and in part on aspects related to the current situation. Third, interpersonal events that may strengthen or disrupt the relationship between client and therapist are viewed as inevitable. Relationships are dynamic entities, within which critical events are bound to take place. The art of therapy is not to try to escape from such events, but to manage them well. The management of interpersonal events depends on the qualities of the fourth variable in the model, which is the therapist. Important therapist qualities involves applying interpersonal reasoning to make sense of and adapt to the ongoing interactions, as well as to be able to use different therapeutic approaches in interaction—in the IRM denoted as “therapeutic modes”—as appropriate to the client's needs in any given situation.

The IRM can be used as a tool for therapists' self-monitoring and subsequent self-reflection. It may serve to enhance the therapist's self-awareness and impact on the way he or she employs the different therapeutic modes, as described in the model. The IRM purports that therapists should aim to increase the repertoire and flexibility in their use of modes, according to the client's needs [1, 2]. In turn, the development and maintenance of a productive therapeutic relationship will logically enhance the quality and effectiveness of therapy.

To be able to cope with the fluctuating job demands in the profession, the occupational therapist needs a certain level of self-efficacy [3]. Generally, self-efficacy describes a person's belief that he or she possesses the competence, knowledge, and skills required to perform the actions leading to a desired outcome [4]. Self-efficacy influences how someone feels, thinks, is motivated, and behaves, and thereby it has an important contribution to self-regulation processes [5]. Applied to the work of occupational therapists, self-efficacy is needed for the therapist's performance in clinical practice, which includes establishing and maintaining therapeutic relationships with a variety of clients [6, 7]. Essentially, the therapeutic relationship should support the client's own occupational engagement as the main agent of change [1]. In that sense, self-efficacy for working with the client-therapist relationship appears to be an important prerequisite for occupational therapists' practice. Unlike other kinds of relationships, where both parties are mutually responsible for building the relationship, the therapeutic relationship is ultimately the therapist's responsibility [1]. Therefore, therapist skills and perceived readiness to employ them are necessary for building and maintaining the therapeutic relationship.

Self-efficacy for therapeutic use of self encompasses the person's perceived competence in performing specific therapeutic actions in a specific situational context [8]. Students, who are often young and may have limited experience of resolving relationship challenges, would therefore generally be considered novices and in need of hands-on guidance and feedback on their performance in such situations. In view of this, the intentional use of the therapeutic relationship has been taught in workshops at two occupational therapy education programs in Norway over the last years [9]. The workshops have aimed to assist the students in building skills related to the therapeutic use of self, and to raise their self-efficacy beliefs concerning their ability to perform these skills in real-life practice [10].

Little is known about the efficacy of such educational efforts. In general, research concerned with the IRM is in a beginning stage, but a recent study showed that occupational therapy students had increased their self-efficacy for therapeutic use of self three months after their participation in an IRM workshop [10]. However, follow-up studies using a longer timeline is needed to examine whether such changes can be sustained over a longer period. Moreover, knowing whether students from different universities develop differently may have educational implications.

*Aim of the Study*. The study aimed to examine the change trajectory in occupational therapy students' self-efficacy for therapeutic use of self during a 10-month period following an introductory IRM workshop. We also aimed to assess whether university program was associated with their change trajectory.

## 2. Method

### 2.1. Design

A longitudinal observational study was conducted. Baseline measurement was performed 2-3 weeks after the introductory workshops (a brief outline of the workshops will follow). The first follow-up was approximately three months after the baseline measurement and the second follow-up approximately 10 months after baseline. A researcher from each of the universities informed the participants about the study and collected the completed questionnaires.

### 2.2. Participants

Students were asked to participate in the study if they were second-year students (third semester) in one of the relevant occupational therapy education programs (Oslo or Trondheim) and provided informed consent to participate. No exclusion criteria were used. Originally, 111 occupational therapy students decided to participate. For the current study, including only those participants with valid scores at each of the three measurement occasions, 89 students (35 from Oslo, 54 from Trondheim) constituted the sample. The participants are described in Table 1. The mean age of the students was 24.5 years (SD = 6.2 years), and female students (*n* = 73, 82.0%) were in vast majority. The students from Oslo were significantly older, compared to the students from Trondheim (*p* < 0.01).

### 2.3. IRM Workshops

The content and organization of the workshops are described in more detail previously [10]. They were conducted in the classroom with the students from both universities. Due to differences between the study programs, the workshop in Oslo had three hours' duration, while the workshop in Trondheim had six hours' duration. There were some differences concerning the pedagogy of the two workshops. However, both included a theoretical introduction to the IRM model, including explanations of its main concepts. Similarly, both workshops included teacher demonstrations, student role-plays using the therapeutic modes, and a concluding plenary discussion based on the students' experiences from the workshop.

### 2.4. Measures

#### 2.4.1. Self-Efficacy for Therapeutic Use of Self

The* self-efficacy for therapeutic use of self* questionnaire was developed by Yazdani and Tune, based on Taylor's [1] original model. The questionnaire consists of three parts, all provided in an appendix to a previous article [10]. Part I asks respondents to rate their level of confidence that they have the required skills to use each of the therapeutic modes. This scale, the* self-efficacy for therapeutic mode use *(N-SETMU; [9]), has been found to have a one-factor structure (factor loadings between 0.68 and 0.81) with good internal consistency between its six items (Cronbach's *α* = 0.82). Part II asks respondents to rate their level of confidence that they have the required skills to recognize client's interpersonal characteristics in therapeutic encounters. This scale, the* self-efficacy for recognizing interpersonal characteristics* (N-SERIC; [6]), was also found to have a one-factor structure (factor loadings between 0.75 and 0.89) with very high internal consistency between its twelve items (Cronbach's *α* = 0.96). Part III asks respondents to rate their level of confidence that they have the required skills to manage the interpersonal challenges that may occur in therapeutic encounters. Echoing the results concerned with the two other scales, the* self-efficacy for managing interpersonal events* scale (N-SEMIE; [11]) was found to have a one-factor structure (factor loadings between 0.72 and 0.84) with very high internal consistency between the items (Cronbach's *α* = 0.94).

#### 2.4.2. Demographic Variables

In addition to the three-part questionnaire, the participants provided information about age (in years) and gender (male = 0, female = 1). All data were collected by self-report.

### 2.5. Data Analysis

All statistical analyses were performed with the IBM SPSS for Windows software, version 24 [12]. Differences in age and gender between students at the two universities were examined with *χ*^2^-tests (gender) and with independent samples* t*-tests (age). Independent* t*-tests were also employed to assess group differences on the outcome variables at each time point. Two-way repeated measures analyses of variance (ANOVA) were used to assess the trajectories of the students' self-efficacy for therapeutic use of self, using university as the between-subjects factor. Gender was included as covariate in a second step of the analysis. In cases of statistically significant interactions with university or gender, analyses would be repeated separately for each of the subgroups. Effect sizes (ES) were provided as partial *η*^2^, and ES > 0.14 was considered a moderate effect size [13]. Adjustments for multiple comparisons were made using the Bonferroni correction. The level of statistical significance was set at *p* < 0.05 and all tests were two-tailed.

### 2.6. Ethics

The study was conducted according to ethical guidelines for research [14]. The participants were informed about the aims and procedures of the study, and written consent forms were provided from all participants. Approval for the study was received from the Norwegian Data Protection Official for Research (project number 49433).

## 3. Results

The multivariate test indicated a significant change in the participants' N-SETMU scores across time, with a large effect size (Wilks' *λ* = 0.56,* F*[2,86] = 33.6, *p* < 0.001, partial *η*^2^ = 0.44), and the time effect showed no statistically significant interaction with university. This result did not vary by gender, but including gender as covariate in the second step of the analysis reduced the effect size of time (partial *η*^2^ = 0.15). The pairwise comparisons revealed that most of the increase in N-SETMU scores occurred between baseline and 3-month follow-up (*M* = 40.5 versus* M* = 44.7, *p* < 0.001), whereas the increase from 3-month to 10-month follow-up was not statistically significant (*M* = 44.7 versus* M* = 46.4, ns).

Similarly, there was a significant change in the participants' N-SERIC scores across time, with a very large effect size (Wilks' *λ* = 0.19,* F*[2,86] = 178.1, *p* < 0.001, partial *η*^2^ = 0.81), and time showed no statistically significant interaction with university. The time effect did not vary by gender, but including gender as covariate in the second step of the analysis reduced its effect size (partial *η*^2^ = 0.34). The quadratic effect of time was also statistically significant and had a large effect size (*F* = 33.0, *p* < 0.001, partial *η*^2^ = 0.28), indicating that the initially increasing curve flattened during the time between 3-month and 10-month follow-up. Similarly, the pairwise comparisons showed that most of the increase in N-SERIC scores occurred between baseline and 3-month follow-up (*M* = 60.9 versus* M* = 80.8, *p* < 0.001), whereas the increase from 3-month to 10-month follow-up was not statistically significant (*M* = 80.8 versus *M* = 84.5, ns).

**Figure 1:**
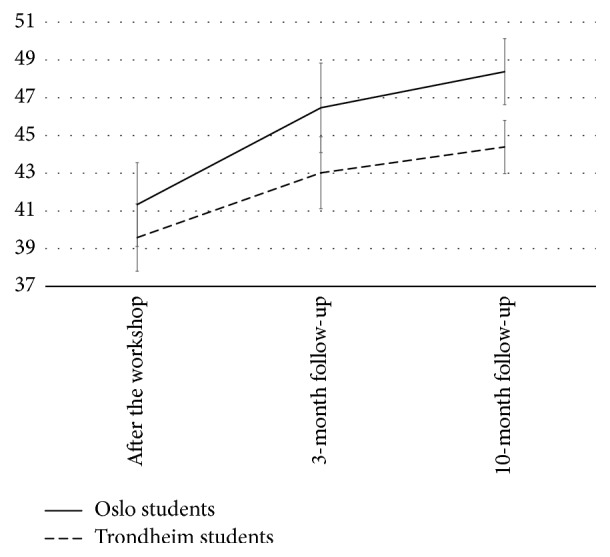
The course of self-efficacy for therapeutic mode use.* Note*. Score range is 6–60, and error bars are calculated by multiplying the standard error of the mean with 1.96. The total sample showed a linear increase in their N-SETMU scores across time.

**Figure 2:**
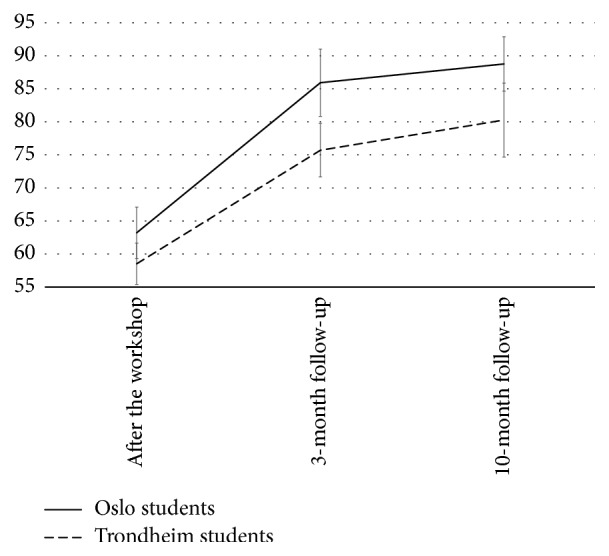
The course of self-efficacy for recognizing client's interpersonal characteristics.* Note*. Score range is 12–120, and error bars are calculated by multiplying the standard error of the mean with 1.96. The total sample showed a linear increase in their N-SERIC scores across time, but also a quadratic trajectory (the flattening curves from 3-month to 10-month follow-up).

**Figure 3:**
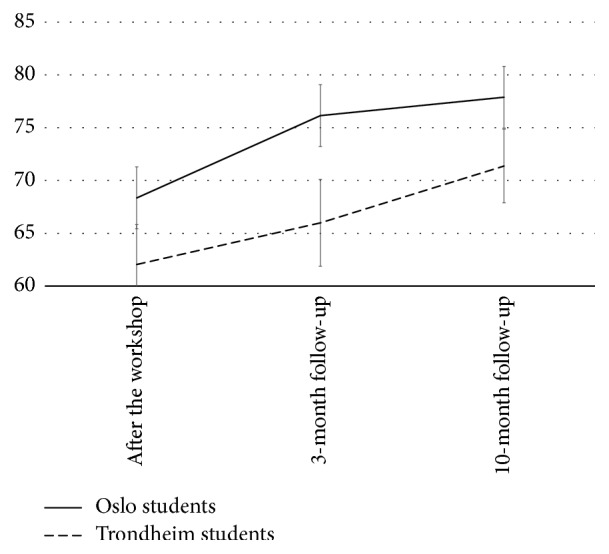
The course of self-efficacy for managing interpersonal events.* Note*. Score range is 11–110, and error bars are calculated by multiplying the standard error of the mean with 1.96. The total sample showed a linear increase in their N-SEMIE scores across time.

The change in the participants' N-SEMIE scores across time was also statistically significant, with a large effect size (Wilks' *λ* = 0.68,* F*[2,86] = 20.1, *p* < 0.001, partial *η*^2^ = 0.32), and the time effect showed no statistically significant interaction with university. This result did not vary by gender, but including gender as covariate in the second step of the analysis reduced the effect size of time (partial *η*^2^ = 0.06). The pairwise comparisons showed that most of the increase in N-SEMIE scores occurred between baseline and 3-month follow-up (*M* = 65.2 versus* M* = 71.1, *p* < 0.001), whereas the increase from 3-month to 10-month follow-up was also borderline and statistically significant (*M* = 71.1 versus* M* = 74.6, *p* = 0.06). Figures 1–3 display the results from the repeated measures ANOVA, controlling for university.

## 4. Discussion

The Intentional Relationship Model (IRM) is a conceptual framework for describing the therapeutic relationship and the therapeutic use of self within occupational therapy practice. It is used to increase the therapist's awareness of the interpersonal aspects of occupational therapy practice, so that therapists can develop and fine-tune skills for clinical interaction [1]. In this study, we investigated occupational therapy students' self-efficacy development related to their therapeutic use of self during a 10-month follow-up period after having attended an IRM workshop at their respective universities. As a whole, the student group significantly increased their self-efficacy during the 10-month follow-up period. Most of this increase in self-efficacy occurred during the first three months, but the increase was sustained across the full 10-month period.

This pattern of self-efficacy development may have occurred because the introduction to the IRM model, as well as working to increase skills in the use of self, was a new experience for the students. Immediately after the workshop, the students may have perceived the new knowledge and skills to be of particular interest. Their increased awareness of the therapeutic relationship and the use of self may have stimulated both intellectual curiosity and further skills development, which in turn may have led to the initial increase in self-efficacy [4]. Moreover, building on H. L. Dreyfus and S. E. Dreyfus' [15] concepts on professional development, the “novice” self-consciously carries out his actions with little reference to the context and perceives the resulting successes and failures more intensely. Thus, the self-conscious beginner may be more prone to experience and report on perceived changes in self-efficacy for new skills, compared to himself at a later point in time. As the students gained more experience during the time between 3-month and 10-month follow-up, they may have started to internalize the new knowledge. According to Dreyfus and Dreyfus [15], this indicates that they would act less self-consciously in a variety of situations and would perhaps not explicitly verbalize and relate their actions to specific concepts as much as they did at first. Hence, they would perhaps no longer be as self-conscious about their own development but may instead have been more oriented towards solving the actual tasks of practice.

The natural progression of the educational course may also play a part in explaining the flattened slope between 3-month and 10-month follow-up. During this time, therapeutic use of self and the students' development in this particular area was no longer explicitly focused in the academic courses [16, 17] and may not have been focused to a similar degree in subsequent practice placements. Thus, the skills and self-efficacy for therapeutic use of self may have been perceived as a less emphasized topic among the students at the 10-month follow-up, and this may have influenced their scores. Finally, the students' mean scores on the outcome scales at 3-month follow-up, particularly among the students from Oslo, are seen as reflecting already high levels of self-efficacy. With already high levels on the relevant scales, it is more difficult to report further increase within the confinements of the scale (“ceiling effect”) [18].

The analyses detected statistically significant change patterns for all outcome measures. However, the change in the N-SERIC showed a considerably larger effect size, compared to the change in other measures. In line with a previous study [10], we note that the N-SERIC measures self-efficacy for skills used in observing and interpreting the interpersonal customs and preferences of the client. Self-efficacy for mode use (N-SETMU) and self-efficacy for managing interpersonal events (N-SEMIE), on the other hand, measure skills for actual behaviors and management of potentially stressful encounters in clinical practice. Therefore, the results suggest that the students' increased their self-efficacy for observation skills in particular. The further development of self-efficacy for actual performing in therapy may depend more on the students' further guided and successful experiences from clinical practice [4].

## 5. Strengths and Limitations

The longitudinal design and the use of participants from two different universities are strengths of the study. In addition, the employed measures have shown good psychometric properties in recent validation studies [6, 9]. However, the participants were relatively few in numbers, and they were recruited by convenience. The sample also represents occupational therapy students from undergraduate programs from one country only. Thus, for several reasons, one should be careful about generalizing the study results.

## 6. Conclusion

This study found that occupational therapy students sustained their initial increase in self-efficacy for therapeutic use of self until 10-month follow-up. In view of the limited focus being placed on this aspect of therapeutic practice during the follow-up period, we consider the results uplifting. The results indicate that students not only improved self-efficacy for therapeutic use of self in the short term; they were also able to sustain the improvements over a longer period. Further studies are needed to assess whether changes are sustained further, and whether student characteristics are associated with favorable trajectories across a longer follow-up period.

## Figures and Tables

**Table 1:** Sample characteristics (*n* = 89).

Variables	All	Oslo	Trondheim	*p*
	*n* = 89	*n* = 35, 39.3%	*n* = 54, 60.7%	
	M (SD)	M (SD)	M (SD)	

*Age *				
Years of age	24.5 (6.2)	27.1 (8.5)	22.9 (3.2)	<0.01

	*n* (%)	*n* (%)	*n* (%)	

*Gender*				
Male	16 (18.0)	6 (17.1)	10 (18.5)	0.87
Female	73 (82.0)	29 (82.9)	44 (81.5)	
